# Hepatoid carcinoma of the pancreas

**DOI:** 10.1186/s12957-015-0586-6

**Published:** 2015-05-20

**Authors:** Po-Chung Kuo, Shih-Chin Chen, Yi-Ming Shyr, Ying-Ju Kuo, Rheun-Chuan Lee, Shin-E Wang

**Affiliations:** Division of General Surgery, Department of Surgery, Taipei Veterans General Hospital, National Yang Ming University, 10 F 201 Section 2 Shipai Road, Taipei, 112 Taiwan; Department of Pathology, Taipei Veterans General Hospital, National Yang Ming University, Taipei, Taiwan; Department of Radiology, Taipei Veterans General Hospital, National Yang Ming University, Taipei, Taiwan

**Keywords:** Hepatoid carcinoma, Pancreas, Pancreatectomy

## Abstract

**Background:**

Hepatoid carcinoma of the pancreas is extremely rare. This article tries to summarize the clinical features and outcomes of pancreatic hepatoid carcinoma.

**Methods:**

The data pool for analysis includes the case we encountered with hepatoid carcinoma of the pancreas and the reported cases in the literature.

**Results:**

Twenty-three cases of hepatoid carcinoma of the pancreas were analyzed. This tumor occurred more frequently in male than in female patients (69.6 vs. 30.4 %). Tumor sizes range from 0.5 to 11.0 cm with median of 6.0 cm. The most common symptom was epigastric pain (36.4 %). When the tumor locates at pancreatic head, nausea/vomiting (62.5 %) is more common, followed by jaundice and epigastric pain (50.0 %). For those at pancreatic body-tail, 42.9 % of the patients presented no symptom. Alpha-fetoprotein (AFP) was abnormally elevated in 60 % of the cases. Hepatoid carcinoma in the pancreas could be either pure form or mixed form with other malignancy (40.9 %), with the most common coexisted pathology of malignant neuroendocrine tumor (22.7 %). Metastasis occurred in 36.4 % of the cases at the diagnosis of this tumor, including liver metastasis in 31.8 % and lymph node metastasis in 21.1 %. The overall 1-year survival rate was 71.1 % and 5-year 40.4 %, with a median of 13.0 months. Unresectability, hepatic, and lymph node metastases are associated with negative impact on survival outcome.

**Conclusions:**

Elevation of serum AFP may be a clue leading to the diagnosis of pancreatic hepatoid carcinoma. This tumor could be mixed form with other malignancy. Surgical resection should be the treatment of choice whenever possible.

## Background

Hepatoid carcinoma is a primary extrahepatic neoplasm resembling hepatocellular carcinoma in terms of morphology and immunohistochemistry and often produces alpha-fetoprotein (AFP) [1–8]. The first case reported by Ishikura et al. in 1985 was described in the stomach [9], while Hruban et al. reported the first case in the pancreas in 1987 [5, 10]. Subsequently, documentation of this unique histopathologic feature has been made in other extrahepatic sites including the esophagus, papilla of Vater, colon, lung, gallbladder, adrenal gland, kidney, urinary bladder, ovary, uterus, vagina, and testicle [1, 5–9, 11–23]. The most common location was stomach, followed by ovary [1–5, 24]. However, hepatoid carcinoma of the pancreas is distinctly rare, and the true incidence is still unknown. In the pancreas, hepatoid carcinoma could be pure form or mixed with other histological components such as neuroendocrine tumor or pancreatic ductal adenocarcinoma. As to the pure hepatocellular features, they have been designated variously as hepatoid carcinoma, hepatoid adenocarcinoma, ectopic hepatocellular carcinoma, hepatoid variant of pancreatic cancer, primary hepatocellular carcinoma of the pancreas, or pancreatic tumor with hepatoid differentiation [11, 12, 23, 25–31]. Like most pancreatic neoplasms, it is often clinically silent until symptoms of obstruction, pain, or bleeding occur. Hepatoid carcinoma of the pancreas has been claimed to have an unfavorable survival outcome because the majority have already metastasized at diagnosis, most frequently to the liver and lymph nodes [5, 9, 11, 19, 22]. Their aggressiveness could derive from a propensity to proliferate in lymphatic and venous vessels mimicking the behavior of hepatocellular carcinoma in the liver [3, 32]. With its rarity and limited experience from sporadic case reports in the literature, the clinical features and behaviors of hepatoid carcinoma of the pancreas have not been clarified so far.

The purposes of this article are to present our clinical experience with hepatoid carcinoma of the pancreas and to analyze an expanded sample size by adding cases from the literature to our pool of study cases. Thus, an attempt is made to clarify the characteristics, clinical presentations, managements, and survival outcomes of this rare tumor.

## Methods

Brief descriptions for the case of hepatoid carcinoma of the pancreas encountered at our institute were made. To clarify the characteristics of hepatoid carcinoma of the pancreas, individualized data of cases with hepatoid carcinoma of the pancreas reported in the English literature were extracted and added to our database to expand the study sample size for a more complete analysis. Two methods were utilized to search for relevant cases in the literature. First, to identify the relevant articles dealing with hepatoid carcinoma of the pancreas in the English literature, a computerized search was performed on the PubMed electronic database, covering data from 1987 to 2014. The following keywords were used for the PubMed search: hepatoid carcinoma of the pancreas, hepatoid differentiation of the pancreas, ectopic liver cancer, and rare pancreas neoplasm. Second, the reference lists of PubMed-selected articles related to hepatoid carcinoma of the pancreas were screened systematically for additional studies of interest. A total of 20 related articles were selected for the study [2–5, 7, 10–12, 23, 25–31, 33–36]. Cases without individualized data or duplicate cases reported in literature were excluded from analysis. The data pool from the related literature and our case were analyzed to determine the characteristics of hepatoid carcinoma of the pancreas including demographics, tumor size, tumor markers, hepatitis markers, clinical presentations, histopathology, treatments, and survival outcomes. These data were also used to make comparisons between two groups: one in pancreatic head and the other one in body-tail.

The statistical analysis was performed using Statistical Product and Service Solutions (SPSS) version 21.0 software (SPSS Inc., IBM, Armonk, NY, USA). All continuous data were calculated using median and mean ± standard deviation (SD) and frequencies, as appropriate to the type of data. The mean values of the continuous variables were compared with a two-tailed Student *t* test. Non-parametric statistical tests were used if the variables did not follow normal distribution. Categorical variables were presented as numbers and percentages. Categorical variables were compared using Pearson’s *χ*^2^ test or Fisher’s exact test contingency tables. The Kaplan-Meier method was used for the calculation of median survival and survival analysis. For all analyses, a *P* value less than 0.050 was considered statistically significant.

## Results

**Fig. 1:**
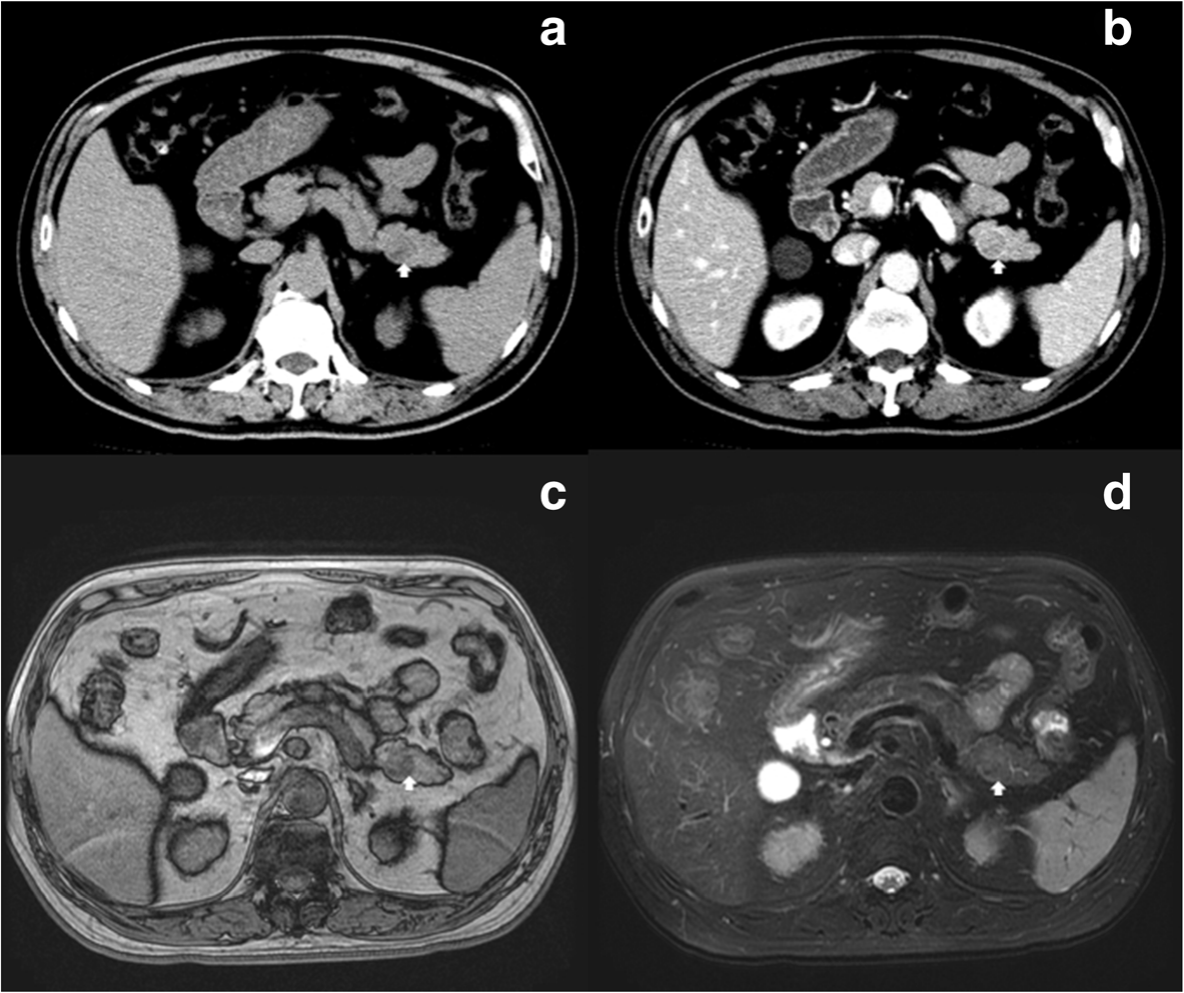
**a** A heterogeneous hypodense nodule (*white arrow*) is depicted at pancreatic tail in pre-contrast CT; **b** After contrast administration, a well-enhanced tumor with pseudocapsule (*white arrow*) is identified; **c** The nodule shows signal drop (fat) on T1 opposed phase MRI image, probably due to fatty metamorphosis (*white arrow*); **d** However, the lesion is isosignal (no water) on T2 weighted MRI image (*white arrow*)

**Fig. 2:**
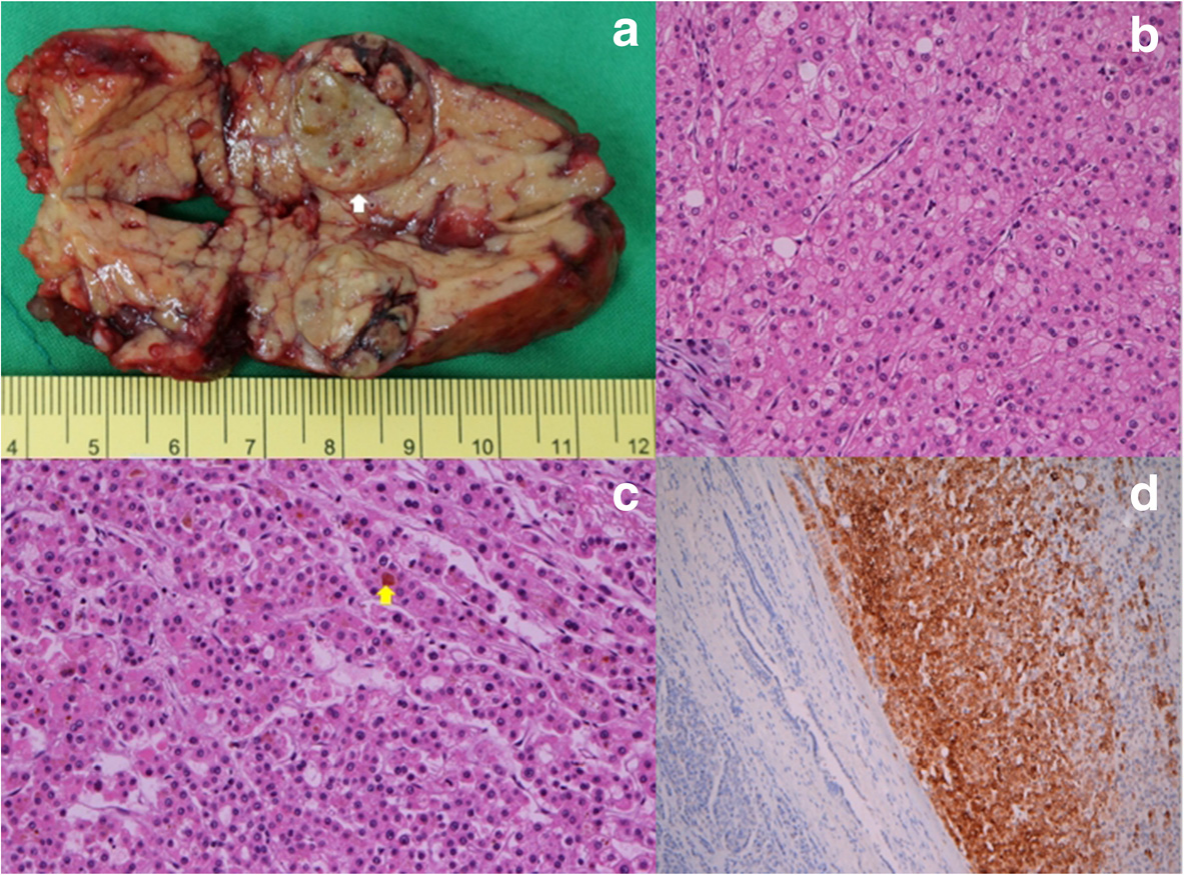
**a** A well-circumscribed with partial encapsulation (*white arrow*), yellow-brown, soft 2 × 2 cm tumor in pancreatic tail; **b** Thick trabeculae of tumor cells, which are polygonal and have abundant eosinophilic granular cytoplasm; **c** Trabecular and acinar growth pattern of the tumor in which bile formation (*yellow arrow*) is also present; **d** Positive staining (*brown in color on the right side*) for hepatocyte antigen

A total of 23 cases of hepatoid carcinoma of the pancreas were collected for this study, including 22 cases from case reports in literature and 1 from our institute. Our case was a 67-year-old male who presented with no symptom but an incidental finding of pancreatic tail mass during survey examinations for recent hypertension. The serum amylase, lipase, and tumor markers including AFP, carbohydrate antigen 19-9 (CA 19-9), and carcinoembryonic antigen (CEA) were all normal. Serum markers for hepatitis B and C were also negative. The image studies showed a 2 × 2 cm heterogeneous hypodense nodule at pancreatic tail in pre-contrast computed tomography (CT) scan. After contrast administration, a well-enhanced tumor with a rim encapsulation was identified (Fig. 1). The nodule showed signal drop (fat) on T1 opposed phase in magnetic resonance imaging (MRI) study, probably due to fatty metamorphosis; however, the lesion was isosignal (no water) on T2 weighted MRI images. The patient underwent robotic distal pancreatectomy with spleen-preservation by Warshaw technique and recovered uneventfully. The cut section of the mass showed a well-circumscribed, yellow to brown, soft, 2 × 2 cm tumor with partial encapsulation (Fig. 2a), resembling the gross features of hepatoma in the liver. Microscopic examination revealed the tumor cells were polygonal with abundant eosinophilic granular cytoplasm and presented with trabecular and acinar growth pattern (Fig. 2b), in which bile formation was also noted (Fig. 2c). The tumor cells were also positive for hepatocyte antigen in histochemical stain (Fig. 2d). He had been followed up for 6 months postoperatively without any signs of recurrence.

Table 1 lists the demographics and clinical presentations of the 23 cases of hepatoid carcinoma of the pancreas. More than half of the cases (60.9 %) were located in pancreatic body-tail. Two cases were diffuse or multifocal, but more prominent in pancreatic head, so they were classified into pancreatic head group for statistical analysis. This tumor occurred more frequently in male patients than in female, 69.6 vs. 30.4 % for total patients, 77.8 vs. 2.2 % for pancreatic head group, and 64.3 vs. 35.7 % for pancreatic body-tail group. The median age was 56 years old, ranging from 21 to 80 years old for overall patients. The median tumor size was 6.0 cm, ranging from 0.5 to 11.0 cm, and there was no significant difference of tumor size between pancreatic head and body-tail groups. Hepatitis B and C were all negative in the reported cases. AFP was abnormally elevated in 60 % of total group, 71.4 % of pancreatic head group, and 50 % of pancreatic body-tail group. Both CA 19-9 and CEA were abnormally elevated in 18.2 % of total patients. For overall patients, the most common symptom was epigastric pain (36.4 %), followed by nausea/vomiting (31.8 %). For pancreatic head group, the most common symptom was nausea/vomiting (62.5 %), followed by epigastric pain and jaundice (50.0 %). For pancreatic body-tail group, 42.9 % presented no symptom and 28.6 % epigastric pain.

**Table 1:** Demographics and clinical presentations of hepatoid carcinoma of the pancreas

	Total	Pancreatic head	Pancreatic body-tail	*P* value
Sex	*n* = 23	*n* = 9	*n* = 14	0.418
Male	16 (69.6 %)	7 (77.8 %)	9 (64.3 %)	
Female	7 (30.4 %)	2 (22.2 %)	5 (35.7 %)	
Age (year)	*n* = 23	*n* = 9	*n* = 14	0.156
Median (range)	56 (21–80)	49 (21–80)	58 (32–79)	
Mean ± SD	53.8 ± 14.9	48.2 ± 17.5	57.4 ± 12.3	
Size (cm)	*n* = 20	*n* = 8	*n* = 12	0.907
Median (range)	6.0 (0.5–11.0)	6.3 (0.5–9.0)	6.0 (1.0–11.0)	
Mean ± SD	5.5 ± 2.7	5.6 ± 2.8	5.5 ± 2.8	
Hepatitis B (+), *n* = 8	0	0	0	N/A
Hepatitis C (+), *n* = 8	0	0	0	N/A
Elevation of tumor markers				
AFP, *n* = 15	9 (60.0 %)	5 (71.4 %)	4 (50.0 %)	0.378
CA 19-9, *n* = 11	2 (18.2 %)	1 (25.0 %)	1 (14.3 %)	0.618
CEA, *n* = 11	2 (18.2 %)	1 (25.0 %)	1 (14.3 %)	0.618
Symptom	*n* = 22	*n* = 9	*n* = 14	
No symptom	7 (31.8 %)	1 (12.5 %)	6 (42.9 %)	0.161
Epigastric pain	8 (36.4 %)	4 (50.0 %)	4 (28.6 %)	0.291
Nausea/vomiting	7 (31.8 %)	5 (62.5 %)	2 (14.3 %)	0.032
Diabetes mellitus	5 (22.7 %)	1 (12.5 %)	4 (28.6 %)	0.308
Jaundice	4 (18.2 %)	4 (50.0 %)	0	0.010
Back pain	4 (18.2 %)	2 (25.0 %)	2 (14.3 %)	0.465
Body weight loss	3 (13.6 %)	1 (12.5 %)	2 (14.3 %)	0.797
Hypoglycemia	1 (4.5 %)	0	1 (7.1 %)	0.636
Skin rash	1 (4.5 %)	0	1 (7.1 %)	0.636
Subcutaneous fat necrosis	1 (4.5 %)	0	1 (7.1 %)	0.636
Gastrointestinal bleeding	1 (4.5 %)	0	1 (7.1 %)	0.636
Night sweating	1 (4.5 %)	0	1 (7.1 %)	0.636
Others	2 (15.4 %)	0	2 (14.3 %)	0.462

*N/A* not available, *AFP* alpha-fetoprotein, *CA 19-9* carbohydrate antigen 19-9, *CEA* carcinoembryonic antigen

Hepatoid carcinoma in the pancreas could be either pure (59.1 %) form or mixed (40.9 %) with other histological findings (Table 2). The most common coexisted pathology was malignant neuroendocrine tumor (22.7 %) including three cases (13.6 %) of nonfunctioning neuroendocrine carcinoma, one malignant glucagonoma (4.5 %), and one malignant insulinoma (4.5 %), followed by ductal adenocarcinoma (9.1 %). Overall, metastasis occurred in 36.4 % of the cases at the diagnosis of this tumor, including liver metastasis in 31.8 % and lymph node metastasis in 21.1 %. Most (85.0 %) of the patients underwent surgical resection.

**Table 2:** Pathology and treatment for hepatoid carcinoma of the pancreas

	Total	Pancreatic head	Pancreatic body-tail	*P* value
Histopathology	*n* = 22	*n* = 9	*n* = 13	0.205
Pure hepatoid carcinoma	13 (59.1 %)	4 (44.4 %)	9 (69.2 %)	
Mixed with other pathology	9 (40.9 %)	5 (55.6 %)	4 (30.8 %)	
Nonfunctioning NEC	3 (13.6 %)	3 (33.3 %)	0	
Ductal adenocarcinoma	2 (9.1 %)	1 (11.1 %)	1 (7.7 %)	
Malignant glucagonoma	1 (4.5 %)	0	1 (7.7 %)	
Malignant insulinoma	1 (4.5 %)	0	1 (7.7 %)	
Nonfunctioning NET	1 (4.5 %)	1 (11.1 %)	0	
Acinar cell carcinoma	1 (4.5 %)	0	1 (7.7 %)	
Metastasis at diagnosis	8 (36.4 %)	2 (22.2 %)	6 (46.2 %)	0.246
Lymph node metastasis	4 (21.1 %)	1 (14.3 %)	3 (25.0 %)	0.525
Liver metastasis	7 (31.8 %)	2 (22.2 %)	5 (38.5 %)	0.372
Treatment	*n* = 20	*n* = 8	*n* = 12	0.670
Surgery Pancreaticoduodenectomy	17 (85.0 %)	7 (87.5 %)	10 (83.3 %)	
Distal pancreatectomy	5 (25.0 %)	5 (62.5 %)	0	
Enucleation	9 (45.0 %)	0	9 (75.0 %)	
Total pancreatectomy	1 (5.0 %)	1 (12.5 %)	0	
Surgery + chemotherapy	2 (10.0 %)	1 (12.5 %)	1 (8.3 %)	
Surgery + TAE	1 (5.0 %)	1 (12.5 %)	0	
Surgery + chemotherapy + TAE	1 (5.0 %)	0	1 (8.3 %)	
Surgery + radiotherapy + TAE	1 (5.0 %)	0	1 (8.3 %)	
No treatment	1 (5.0 %)	0	1 (8.3 %)	
Other	2 (10.0 %)	1 (12.5 %)	1 (8.3 %)	
	1 (5.0 %)	0	1 (8.3 %)	

*NEC* neuroendocrine carcinoma, *NET* neuroendocrine tumor, *TAE* transarterial embolization

The overall 1-year and 5-year survival rates were 71.1 and 40.4 % respectively, with a median of 13.0 months and a mean of 18.1 ± 21.8 months (Table 3). Metastasis, either liver or lymph node metastasis, has negative impact in survival outcome. Prognosis in tumor resection group was significantly better than non-resection group. Long-term survival is possible only in the resection group with 53.5 % 1-year and 53.5 % 5-year survival, and all the patients without resection died within 1 year after diagnosis. Tumor location (head vs. body-tail) and histopathology (pure vs. mixed form) did not play a significant role in predicting the survival outcome.

**Table 3:** Survival and prognosis for hepatoid carcinoma of the pancreas

	Case *n*	Median (range)	Mean ± SD	1-year survival	5-year survival	*P* value
Total	22	13.0 (2.0–101.0)	18.1 ± 21.8	71.1 %	40.4 %	
Location						0.780
Pancreatic head	9	8.0 (3.0–48.0)	14.3 ± 14.8	77.8 %	29.2 %	
Pancreatic body-tail	13	14.0 (2.0–101.0)	20.7 ± 25.9	51.3 %	51.3 %	
Histopathology						0.763
Pure hepatoid carcinoma	13	12.0 (2.0–48.0)	15.5 ± 13.3	56.1 %	56.1 %	
Mixed hepatoid carcinoma	8	11.0 (2.8–101.0)	22.7 ± 32.9	75.0 %	25.0 %	
Metastasis						0.001
No	13	15.0 (4.0–48.0)	18.5 ± 12.3	88.9 %	59.3 %	
Yes	8	4.5 (2.0–101.0)	18.9 ± 33.8	25.0 %	12.5 %	
Lymph node metastasis						<0.001
No	15	15.0 (4.0–101.0)	23.7 ± 24.5	83.3 %	62.5 %	
Yes	4	4.5 (2.0–12.0)	5.8 ± 4.5	0	0	
Liver metastasis						0.003
No	15	14.0 (4.0–48.0)	17.2 ± 11.9	80.8 %	53.9 %	
Yes	7	3.0 (2.0–101.0)	20.0 ± 36.4	28.6 %	14.3 %	
Resection of tumor						<0.001
Yes	17	15.0 (2.0–101.0)	21.8 ± 23.6	53.5 %	53.5 %	
No	3	6.0 (3.0–12.0)	7.0 ± 4.6	0	0	

## Discussion

Hepatoid means “liver-like”. Therefore, hepatoid carcinoma often refers to a tumor-like primary hepatocellular carcinoma in the liver but extrahepatic location. These tumor cells have characteristic features similar to those of hepatocellular carcinoma. Microscopically, hepatoid carcinoma is composed of cords of polygonal cells with abundant, eosinophilic cytoplasm and centrally located nuclei in the sheet-like or trabecular portions, occasionally featuring bile production and/or bile canaliculi formation, and together with elevated serum levels of AFP in most reported cases but not in all [3, 5, 15, 28, 35]. Tumor cells may show one or a mixture of trabecular, medullary, and glandular patterns, and varying degrees of differentiation, ranging from well-differentiated with a morphology typical of hepatocytes to less differentiated and irregular forms [3].

The pathogenesis of hepatoid carcinoma of the pancreas is not completely understood. There are two theories to explain the histopathological features of this unique tumor. One is “ectopic liver” theory from the point of view of embryology. Since the pancreas and liver cells are both derived from the foregut endoderm, ectopic liver tissue probably exists in the pancreas. This theory of common embryologic origin appears to be the most widely accepted pathogenetic mechanism of the extrahepatic hepatoid carcinoma. It is believed that activation of the process of liver-specific carcinogenesis could also occur in the ectopic liver tissue in the pancreas [1, 2, 11, 30, 37]. The reported incidence of ectopic liver tissue in different organs ranges between 0.24 and 0.47 % [37, 38]. Ectopic liver tissue is also reported in several sites, such as the stomach, gallbladder, hepatic ligaments, omentum, retroperitoneum, and thorax where hepatoid carcinoma has been reported [1, 11, 30, 37]. These evidences suggest that hepatoid carcinoma could originate from ectopic liver tissue in the pancreas. The other histogenic mechanism is “pancreas-to-liver transdifferentiation” theory, which might be related to multipotent/stem cells in the pancreas. Hepatocyte function following the transdifferentiation has been demonstrated in the animal studies as the pancreas of adult mice contains hepatocyte progenitor cells capable of significant therapeutic liver reconstruction [39, 40]. It is claimed that pancreatic multipotent/stem cells normally suppress hepatocytic differentiation genes, which could be activated during carcinogenesis or under particular unknown environmental conditions [1, 28, 33, 35, 36]. Hepatocyte transdifferentiation from native pancreatic ductal, acinar, or intermediate cells has been documented in animal models following exposure to carcinogens and copper depletion [4, 23]. This theory might provide an explanation for and could be supported by the pathological findings in mixed hepatoid tumors in the pancreas [4]. So far, no association of hepatitis B and C with pancreatic hepatoid carcinoma was reported by this study. It implies that viral hepatitis might not play a significant role in the pathogenesis of this extrahepatic hepatoid carcinoma.

The median age of reported patients was 56 years (range 21–80), about 7 years less than the age of patients with hepatocellular carcinoma and 15 years less than the age of patients with pancreatic ductal adenocarcinoma. Gender was predominant in male patients (69.6 %), which was similar to that of hepatocellular carcinoma, instead of pancreatic ductal adenocarcinoma [2, 10, 26]. More than half of the cases of this tumor (60.9 %) were located in pancreatic body-tail by this study, whereas approximately 75 % of all pancreatic carcinomas occur within the head or neck of the pancreas, 15–20 % occur in the body of the pancreas, and 5–10 % occur in the tail [41]. Therefore, many cases, 31.8 % in this study, were asymptomatic and incidentally discovered. Overall, the most common presenting symptom was epigastric pain (36.4 %), followed by gastrointestinal upset with nausea and/or vomiting (31.8 %). However, when the pancreatic hepatoid carcinoma was in pancreatic head, jaundice was nevertheless a common clinical presentation (50 %), similar to pancreatic head adenocarcinoma. Elevation of serum AFP level was often present at time of diagnosis [1, 8, 12, 14, 15, 18, 19, 23, 26, 31, 42, 43], as also shown in 60 % of the cases of this study. On the contrary, serum CA 19-9 and CEA were not a good diagnostic tool for this tumor because these two tumor markers were not usually elevated. Interestingly, this study showed that many (40.9 %) of the pancreatic hepatoid carcinomas could be mixed with other tumor components such as nonfunctioning as well as functioning neuroendocrine tumors, pancreatic duct adenocarcinoma, and even rare acinar cell carcinoma which might present subcutaneous fat necrosis [5].

The main differential diagnosis of (primary) hepatoid carcinoma of the pancreas would be secondary (metastatic) hepatocellular carcinoma from the liver. Since these two tumors share numerous clinicopathological features such as morphology, immunohistochemistry, and elevated serum AFP, differential diagnosis between the primary and secondary could be very challenging [1, 3]. Clinically, the incidence of hepatocellular carcinoma spreading to the pancreas is low, only 2.7–5.6 %, and the metastasis is usually a late finding [1, 44]. Long-term follow-up is needed to exclude the possibility that the pancreatic tumor represents a metastasis from the liver [1]. If bile production cannot be identified histologically, then the differential diagnosis should also include other primary pancreatic tumors with large eosinophilic tumor cells, such as intraductal oncocytic papillary neoplasms, pancreatoblastoma, poorly differentiated pancreatic adenocarcinoma, islet cell tumors, and acinar cell carcinoma, but should be easily distinguished by histological examinations for their own characteristics of morphology and immunohistochemistry [1, 3–5, 28].

With limited experience due to its rarity, the treatment approach is far from being standardized. Nevertheless, 85 % of reported cases underwent surgery. Radical surgical resection seems to be the best treatment for disease-free survival [3–5, 11, 12, 23, 25, 27, 30, 33, 34, 36, 42]. It suggests that surgical resection be the treatment of choice, whenever possible, and complete resection of the tumor appears to be the best option. The roles of other postoperative treatment modalities are still unclear [1–3]. A certain degree of response to chemotherapy with long-term survival has been reported in locally unresectable, metastatic or recurrent disease [23, 42]. Therefore, it implies that aggressive treatment could be warranted even in the case of locally advanced diseases and resection of metastases might be considered as well [1, 23, 28, 42].

Survival outcomes of the pancreatic hepatoid carcinoma are variable in reported cases and appear unclear due to its rarity and possible heterogeneity [1, 3, 26, 34]. Our study shows that overall 1-year survival rate is 71.1 % and 5-year 40.4 %, with a median of 13.0 months and a mean of 18.1 ± 21.8 months. Neither tumor location (head vs. body-tail) nor histopathology (pure vs. mixed pancreatic hepatoid carcinoma) plays a role in the prognosis. Presence of metastasis, either lymph node or liver, would predict poor survival outcome. Long-term survival is possible only in the resection group, with 53.5 % 1-year and 5-year survival after resection. The median survival (6.0 months) for the unresectable group is similar to that for unresectable pancreatic adenocarcinoma. However, the natural history and prognosis of the disease could not be accurately predicted with limited data.

## Conclusions

Hepatoid carcinoma of the pancreas is an extremely rare tumor. Elevation of serum AFP may be a clue leading to the diagnosis of this tumor; however, preoperative diagnosis is still challenging since there is no characteristic clinical features and, therefore, diagnosis is usually made based on specific histological findings after resection. No association of hepatitis B and C with pancreatic hepatoid carcinoma has been reported so far. This tumor could be mixed with other more common pancreatic tumors, such as neuroendocrine tumors and even ductal adenocarcinoma. Surgical resection should be the treatment of choice whenever possible since long-term survival is possible only in the resection group. Presence of lymph node or liver metastasis would predict poor survival outcome.

## Abbreviations

AFP: Alpha-fetoprotein
CA 19-9: Carbohydrate antigen 19-9
CEA: Carcinoembryonic antigen
CT: Computed tomography
MRI: Magnetic resonance imaging
